# Fungal Symbionts Enhance N-Uptake for Antarctic Plants Even in Non-N Limited Soils

**DOI:** 10.3389/fmicb.2020.575563

**Published:** 2020-10-23

**Authors:** Ian S. Acuña-Rodríguez, Alexander Galán, Cristian Torres-Díaz, Cristian Atala, Marco A. Molina-Montenegro

**Affiliations:** ^1^Laboratorio de Biología Vegetal, Instituto de Ciencias Biológicas, Universidad de Talca, Talca, Chile; ^2^Centro de Investigación en Estudios Avanzados del Maule (CIEAM), Vicerrectoría de Investigación y Postgrado, Universidad Católica del Maule, Talca, Chile; ^3^Departamento de Obras Civiles, Facultad de Ciencias de la Ingeniería, Universidad Católica del Maule, Talca, Chile; ^4^Centro Regional de Estudios Ambientales (CREA), Universidad Católica de la Santísima Concepción, Concepción, Chile; ^5^Laboratorio de Genómica y Biodiversidad (LGB), Departamento de Ciencias Naturales, Universidad del Bío-Bío, Chillán, Chile; ^6^Laboratorio de Anatomía y Ecología Funcional de Plantas (AEF), Instituto de Biología, Pontificia Universidad Católica de Valparaíso, Campus Curauma, Valparaíso, Chile; ^7^Centro de Estudios Avanzados en Zonas Áridas (CEAZA), Facultad de Ciencias del Mar, Universidad Católica del Norte, Coquimbo, Chile

**Keywords:** plant-fungi interactions, nitrogen, endophytes, Antarctic vascular plants, ornithogenic soils

## Abstract

Plant-fungi interactions have been identified as fundamental drivers of the plant host performance, particularly in cold environments where organic matter degradation rates are slow, precisely for the capacity of the fungal symbiont to enhance the availability of labile nitrogen (N) in the plant rhizosphere. Nevertheless, these positive effects appear to be modulated by the composition and amount of the N pool in the soil, being greater when plant hosts are growing where N is scarce as is the case of Antarctic soils. Nevertheless, in some coastal areas of this continent, seabirds and marine mammal colonies exert, through their accumulated feces and urine a strong influence on the edaphic N content surrounding their aggregation points. To evaluate if the fungal symbionts (root endophytes), associated to the only two Antarctic vascular plants *Colobanthus quitensis* and *Deschampsia antarctica*, act as N-uptake enhancers, even in such N-rich conditions as those found around animal influence, we assessed, under controlled conditions, the process of N mineralization in soil by the accumulation of NH_4_^+^ in the rizhosphere and the biomass accumulation of plants with (E+) and without (E−) fungal symbionts. Complementarily, taking advantage of the isotopic N-fractionation that root-fungal symbionts exert on organic N molecules during its acquisition process, we also determined if endophytes actively participate in the Antarctic plants N-uptake, when inorganic N is not a limiting factor, by estimating the δ^15^N isotopic signatures in leaves. Overall, symbiotic interaction increased the availability of NH_4_^+^ in the rhizosphere of both species. As expected, the enhanced availability of inorganic N resulted in a higher final biomass in E + compared with E− plants of both species. In addition, we found that the positive role of fungal symbionts was also actively linked to the process of N-uptake in both species, evidenced by the contrasting δ^15^N signatures present in E+ (−0.4 to −2.3‰) relative to E− plants (2.7–3.1‰). In conclusion, despite being grown under rich N soils, the two Antarctic vascular plants showed that the presence of root-fungal endophytes, furthermore enhanced the availability of inorganic N sources in the rhizosphere, has a positive impact in their biomass, remarking the active participation of these endophytes in the N-uptake process for plants inhabiting the Antarctic continent.

## Introduction

In cold environments as polar and alpine regions the edaphic nitrogen is mainly available as organic compound, imposing metabolic restrictions to the biological mineralization of nitrogen (Shaver and Chapin, 1980; Pietr et al., 1983; Atkin, 1996). To cope with the inorganic N scarcity, plants take advantage of symbiotic interaction with microorganisms (e.g., root mycorrhizal symbionts and root endophytes), as a strategy to enhance their nutritional status (Hobbie et al., 2000; Newsham, 2011; Acuña-Rodríguez et al., 2020). The benefit of the interactions, as described in plant-mycorrhyzae interactions from the Arctic tundra (Hobbie and Högberg, 2012), are related to the capacity of the microbial symbionts to mineralize complex organic N compounds into inorganic forms like ammonium (NH_4_^+^) and nitrate (NO_3_^–^), which are easily absorbed by the plant’s roots. In consequence, the plant-microorganisms association increases N acquisition and enhances the ecophysiological performance of plants (Hobbie and Hobbie, 2008).

The microbiome associated with the Antarctic vascular flora is dominated by the ascomycetous fungi known as dark septate endophytes or DSE (Upson et al., 2009b; Newsham, 2011; Ruotsalainen, 2018). These symbiotic fungi, usually found in the roots, can enhance plant nutrient acquisition, particularly N and P (Newsham, 2011; Hill et al., 2019). However, the shift from organic to inorganic N as nutrient source seems to alter the effect of some root DSE in their host plants, either positively or negatively. This is similar to what has been found for the plant-mycorrhizae interaction of the Arctic tundra in which the role of mycorrhizae on the net plant N-uptake decrease if inorganic N become more available (Hobbie et al., 2000; Johnson et al., 2010). As shown by Upson et al. (2009a), under controlled conditions, four out of six DSE strains had positive effects on shoot and root biomasses of *Deschampsia antarctica* (Poaceae) individuals only when grown using organic N as nutrient source. When supplied with inorganic N, some detrimental effects on the plant were observed (Upson et al., 2009a), presumably because both plant and fungi compete for soil resources, shifting the plant-DSE association from beneficial to negative for the host. Thus, the positive role of DSE root-symbionts on their host plants’ performance is still not conclusive and appears to be highly dependent on the environmental conditions (Newsham, 2011; Acuña-Rodríguez et al., 2020).

Among the terrestrial ice-free areas that allow the life of vascular plants in Maritime Antarctic, those that harbor ornithogenic soil, represent a particular edaphic environment due to their extremely high N concentration (Pires et al., 2017). During the summer, the animal N input produces a patchy spatial distribution of edaphic N, which concentrates around colonies (Bölter et al., 1997; Park et al., 2007). For example, it has been estimated that in Maritime Antarctica, total soil N could vary from highly enriched (N_tot_ = 14.9–8.8 g kg^–1^) surrounding animal colonies, to highly depleted (N_tot_ = 0.5–0.17 g kg^–1^) approximately 800 m away from the colony’s influence (Bölter et al., 1997; Łachacz et al., 2018). Furthermore, the composition of the N pool can also vary drastically depending on the distance to these colonies. The rapid mineralization of animal urea not only raise local ammonium^+^ concentrations in the presence of water, but also produces a volatile N source through the emanation of gaseous ammonia (Pietr et al., 1983), which can be exported up to 1 km away from the bird colonies, depending on the local topography and wind dynamics (Erskine et al., 1998; Bokhorst et al., 2019a). This inorganic N input, spontaneously mineralized from animal-originated N-forms, has been related to the greater performance of lowland coastal plant populations compared with those from more inland locations (Androsiuk et al., 2015). Thus, given that the composition of the N-pool (i.e., organic or inorganic) is known to alter the effect of microbial symbiotic on plants (beneficial or costly), it can be predicted that in ornithogenic N-enriched soils, N-acquisition by Antarctic vascular plants might not be exclusively attributed to the role of symbiotic microorganisms.

Several studies have tested this hypothesis using the isotopic fractionation that occurs during the biological N mineralization in some fungal symbiont-plant associations (Benavent-González et al., 2019 and references therein). Given the natural existence of two stable isotopes of nitrogen (^14^N and ^15^N), the proportion of the heavier isotope in both the N source (soil) and N products (i.e., plant and fungal tissues), has been proposed to be affected by the active role of fungal symbionts in the process of N uptake (Högberg, 1997). For example, during the acquisition of organic N mycorrhizal fungi is prone to retain ^15^N-enriched N, while ^15^N-depleted N is transferred to the plant hosts (reviewed in: Hobbie and Högberg, 2012). Hence, in this plant-fungi interaction model, the intermediate step of acquiring N through the fungal symbiont generates low δ^15^N values in foliar tissues compared to the isotopic signature of the soil N source (Michelsen et al., 1998; Hobbie et al., 2000). Nevertheless, unlike most plant communities, the microbiota associated to the roots of the Antarctic plants is dominated by DSE instead of mycorrhizal fungi (Upson et al., 2009b). Antarctic endophytes and mycorrhizal fungi, however, seem to play a similar ecological role enhancing nutrient acquisition and N in particular (Hill et al., 2019; Acuña-Rodríguez et al., 2020).

The main goal of the present study was to explore the role of fungal endophytes on the N biological mineralization and plant N-acquisition processes when inorganic N is not limiting. We specifically addressed two questions: (i) is the organic N-mineralization in the rhizosphere of two vascular Antarctic plants enhanced by the presence of root endophytes under N-enriched conditions? and (ii) does root endophytes participate in the N-uptake of these plant species when inorganic N is not a limiting factor? To answer these questions we specifically measured: (a) the percentage of NH_4_^+^ accumulated in the soil through time in plants inoculated and non-inoculated with root fungal endophytes to determine the relevance of this symbiotic association on the process of N biological mineralization, (b) the differences in biomass accumulation between those inoculated and non-inoculated plants and (c) the δ^15^N isotopic signature in foliar tissues, we determined if fungal endophytes actively participate in the process of N-acquisition when inorganic N is not a limiting factor. By answering these questions, we are able to evaluate if the root fungal endophytes maintain their positive role as N-uptake enhancers for their hosting plants when grown in N-rich ornithogenic soils, such as those found in some Antarctic habitats.

## Materials and Methods

### Sampling Site and Plant Material

**FIGURE 1:**
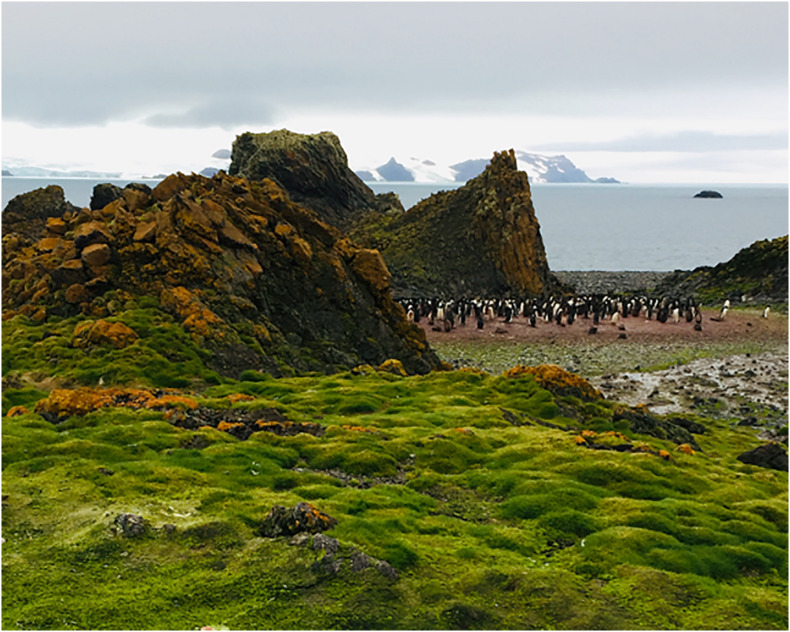
Antarctic plant community growing near a big penguin colony in the study site (Admiralty bay, King George Island, South Shetland, Antarctica).

Healthy individuals of *C. quitensis* and *D. antarctica* (*n* = 30 per species) were collected along with their rhizospheric soil from populations located in the western coast of Admiralty Bay, King George Island, Southern Shetlands, Maritime Antarctica (Figure 1). We focused our sampling on those individuals inhabiting microhabitats surrounding colonies of marine birds and mammals (mostly Gentoo penguin, *Pygoscelis papua*) present along the shore (Bölter, 2011; Figure 1). As most of these ornithogenic coastal soils, the sampled sites had primarily rocky-sandy substrates with a marked presence of coarse skeletal fractions and incipient stratification (Bölter, 2011). Collected plants were carefully put in plastic containers and transported from the field to the laboratory within 2 days, trying to avoid plant stress due to drought or extreme temperatures. Once in the laboratory, all plants were maintained at 5°C in an automatic air-cooling growth chamber (model: LTJ300LY; Tianyi Cool, China), and at a constant photosynthetic photon flux density (PPFD) of 240 μmol m^–2^ s^–1^ in daily photoperiods of 19/5 h light/dark to simulate the study site environmental conditions during the austral growing season.

### Production of Axenic (E−) and Inoculated (E+) Plants

After 2 weeks of acclimation, plants from each species were vegetativelly propagated. Five tillers from 10 field-collected individual were separated, rinsed with distilled water, and treated with a 1 h submersion in 2 g l^–1^ of Benlate^®^ (benomyl [methyl [1-butylamino carbonyl]-1H-benzimidazol-2-yl] carbamate (DuPont, Wilmington, United States) at room temperature. The resulting 50 tillers per species were transplanted to 50 cc cells pot-in-frame in a speedling tray. Cells were previously filled with autoclavated soil from the study site. The selection of the fungicide was based on its broad spectrum of action, low leaching rates (Rhodes and Long, 1974), and because it is harmless to Antarctic plants, as it has been observed in previous experiments made by our research group (Ramos et al., 2018; Barrera et al., 2020; Hereme et al., 2020). After 4 weeks, endophyte infection was assessed by counting aniline blue-stained fungal hyphaes in root cross-sections in 10% of the produced plants as the percentage of infested root length (Bacon and White, 2000). Complementarily, sterilized root fragments from the selected individuals were plated on Petri dishes containing potato dextrose agar (PDA, Difco, United States) plus chloramphenicol at 100g ml^–1^ and were incubated for a 30-days at 18°C. Only those plants that showed <5% of infested-root length and no outgrowth of fungi into the PDA media were considered as “fungal endophyte-free” (E−), becoming suitable for their use in the subsequent experiment. Until the beginning of the experiment, E− tillers were sprinkled once a week during this process with the same Benlate solution (2 g l^–1^, see above) to extend the time of the axenic state.

Half of the obtained E− individuals were re-inoculated with fungal spores from the most abundant root fungal endophyte reported for the studied populations of each plant species; these correspond to *Penicillium chrysogenum* (strain AFE001, Genebank Accession Number: KJ881371) in *C. quitensis* and *Penicillium brevicompactum* (strain AFE002, GeneBank Accession Number: KJ881370) in *D. antarctica* (Molina-Montenegro et al., 2016). In each case, the inoculum consisted of a concentrated mix of spores (5,000 spores g^–1^) obtained from stored cultures of the referred fungal strains that are routinely maintained at the laboratories of the Instituto de Ciencias Biológicas, Universidad de Talca^1^. The liquid inoculum-mix was added three times during a week (10 ml per individual) to ensure fungal association. Two weeks after the first inoculation, occurrence of effective symbiosis was corroborated by routine staining and microscopic observation in three randomly selected individuals from each species (Supplementary Figure 1). The resultant endophyte free (E−, *n* = 20 for each species) and endophyte free, but reinoculated (E+, *n* = 19 for *C. quitensis*; 18 for *D. antarctica*) individuals, were then transplanted to 300 ml pots filled with sterilized Antarctic soil. We conducted a previous verification of the soil microbiological condition by cultivation of a subsample of the sterilized Antarctic soil on PDA plates where after 2 weeks no fungal growth was subsequently observed. The experiment lasted for 60 days. During that time, all plants were maintained in the same light conditions (PFD of 240 μmol m^–2^ s^–1^ in a 19/5 h light/dark day), and 40 ml of tap water were added to each plant every week.

### Role of the Plant Symbiont on Soil N Mineralization

To estimate if fungal endophytes participate in the mineralization of organic N, we compared the percentage of ammonium (NH_4_^+^) in the rhizospheric soils from E+ and E− individuals (*n* = 7/fungal treatment) of each species prior to the transplant, and after 7, 15, 30, and 60 days of experiment. The substrate used for plant growing was obtained from 15 soil samples of 1 kg (1–5 cm depth) collected near Arctowski station (Antarctica) in a zone without Penguin colony influences. Those soil samples were homogenized before measuring their total N content (*n* = 3, N content = 7.8 ± 0.8 g of N kg^–1^). After being autoclavated, the substrate was enriched with an organic N source (urea) to mimic the average N condition (∼16 g N kg^–1^) described for the local coastal soils around penguin colonies, which represents an enriched N condition for Antarctic soils (Kozeretska et al., 2010). We used urea because it is an intermediate compound in the degradation pathway of uric acid, which is heavily deposited in soils close to coastal colonies of birds and mammals (Pietr et al., 1983), and because despite its spontaneous degradation at acidic conditions in the presence of water, it can be mineralized by other fungal endophyte species (Jumpponen et al., 1998).

Soil sampling from each experimental plant focused on the soil material around the roots by a careful removal of the plant from its pot. For the total N estimation, the Kjeldahl digestion method was used (Allen, 1989). Briefly, a 0.2 g soil sample was added to 0.05 g of catalyst (Li_2_SO_4_:CuSO_4_ in 10:1 ratio) and 1 ml of a digestion reagent (33 g of C_7_H_6_O_3_ in 1 l of H_2_SO_4_) in a digestion tube, and then further heated to 370°C in a digestion block until the solution was clear (∼ 6 h). The cooled digested soil sample was diluted in 10 ml of distilled water, filtered (Whatman filter paper N°44), and then diluted in 50 ml of distilled water. Flame atomic absorption spectrometry was finally used to determine the individual element concentrations. Ammonium was also determined by the colorimetric analysis of 5 g of air-dried soil samples immersed on 50 ml of 2 M KCl for 30 min and filtered through filter paper (Whatman N° 42) (Knepel, 2003), using a continuous flow injection analyzer (FIAflow2, Burkard Scientific, Uxbridge, United Kingdom). Nitrogen mineralization was then estimated to 7, 15, 30, and 60 days after the beginning of the experiment in the soil from pots containing E+ and E− individuals of both species as the relative N-NH_4_^+^ content (%) compared with the initial concentration observed in the soil substrate at day 0. Since thermal soil sterilization may affect nutrient availability, soil samples were tested for differences in total N in sterilized and non-sterilized soil samples (*n* = 5) prior to being enriched for experimentation, and no statistical differences were found between them (*t*-test = 0.93; *p* = 0.77).

### Effect of DSE on Plant N Uptake

To determine the participation of fungal endophytes on the process of plant N-uptake, we estimate the foliar δ^15^N signature of E+ and E− individuals at day 60 (*n* = 10 per species) and compare their patterns of ^15^N isotopic discrimination with respect to the initial soil substrate. To calculate the latter, we estimated the δ^15^N signature in five substrate samples (δ^15^N = 8.8 ± 0.52‰), and five samples with the added urea (δ^15^N = −1.46 ± 0.02‰). Then, the final value of the experimental substrate (δ^15^N = 3.67) was calculated as:

δN15=t⁢o⁢t⁢a⁢l(δN15×s⁢o⁢i⁢l[N]+s⁢o⁢i⁢lδN15×u⁢r⁢e⁢a[urea])/[N]t⁢o⁢t⁢a⁢l

The δ^15^N isotopic ratios were assessed in the Laboratory of Biogeochemistry and Applied Stable Isotopes at the Pontificia Universidad Católica de Chile (Santiago, Chile) using an Isotope Ratio Mass Spectrometer, IRMS (Thermo Delta Advantage) coupled to an Elemental Analyzer (Flash EA2000). Stable isotope abundances were expressed in δ-notation as the deviation from standards in parts per thousand (‰) obtained from:

δN15=1000×[(R/s⁢a⁢m⁢p⁢l⁢eR)s⁢t⁢a⁢n⁢d⁢a⁢r⁢d-1]

where *R* is the corresponding ^15^N/^14^N ratio for either a given sample or the atmospheric N_2_ standard for ^15^N isotopic fractionation (Hobbie et al., 1999). The analytical precision of the isotopic measurements of multiple replicate analyses was 0.2‰. Complementarily, to estimate the overall effect of the symbiosis on the plant individual performances, the total dry biomass of 10 E+ and 10 E− plants per species was estimated at the end of the experiment. All tissues (included fallen leaves) were oven-dried at 70°C for 72 h and weighted with an electronic precision balance (Boeco BBl-54, Germany).

### Data Analysis

We used General Additive Mixed Models (GAMMs) to evaluate in each species the shape of the temporal trend of the edaphic NH_4_^+^ concentrations, and the potential effect that the infection status (E + and E−) can exert on its direction. Using the “gamm” function from the *mgcv* R-package v.1.8.32 (Wood, 2017), we modeled the soil NH_4_^+^ content along time in response to the infection status of the plants by fitting a smoothed spline to the data according to the following equation:

y=i⁢jα+0αi1⁢knfectionstatus+kf(day,i⁢jinfectionstatus)ki⁢j⁢k+εi

Where the response of the *i*th individual at the *j*th day (*y*_*ij*_) is defined by the model intercept (α*_0_*), the difference between α*_0_* and the mean response of the respective infection status *k* (α*_1*k*_*), the smooth temporal function by infection status *k, f_*ijk*_*, and the individual error (ε_*i*_), which is assumed to be a random factor with a Gaussian distribution *ε_*i*_* ∼ *N*(0, σ^2^). In this sense, within each species a fitted spline an its approximated 95% confidence interval was calculated for each experimental group (E+ or E−). In addition, the final average NH_4_^+^ content in soils, the δ^15^N isotopic values at day 60, and the average final dry biomasses were all analyzed using a two-way ANOVA’s including endophyte treatment (E+ or E−) and the species of host plant as fixed factors. For the *post-hoc* contrast of treatments between species, the Honest Significant Difference (HSD) test of Tukey was applied on the two-way ANOVA outputs from the final biomass and δ^15^N isotopic signature datasets. All statistical analyses were carried out in the R Language and Statistical Environment v3.6.2 (R Core Team, 2019), after testing for normality and homogeneity of variances assumptions using the Shapiro-Wilks and Bartlett tests, respectively.

## Results

**FIGURE 2:**
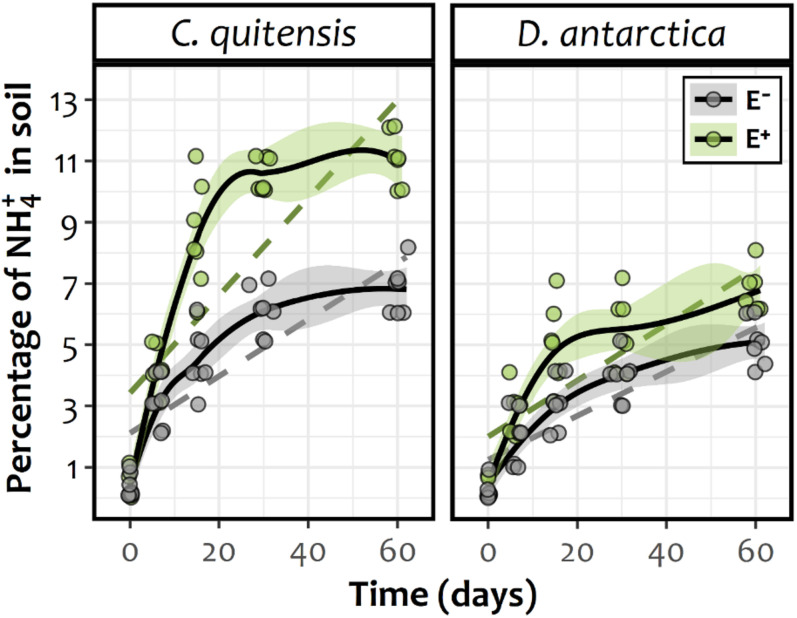
Temporal smooth functions (solid lines) and their approximate 95% confidence intervals (shaded area) for E− (gray) and E+ (green) individuals of either Antarctic vascular plant species *Colobanthus quitensis* or *Deschampsia antarctica*, as derived from a GAMM fitted model. Confidence interval overlapping can be considered to dilute any significant difference between splines during the respective time. As a reference, dashed lines represent the simplest (i.e., linear) model fit for each experimental group. The respective coefficients were, for *C. quitensis*: slope E− = 0.093, SE E− = 0.015, df E− = 66; slope E+ = 0.161, SE E+ = 0.016, df E+ = 66 and for *D. antarctica*: slope E− = 0.071, SE E− = 0.009, df E− = 66; slope E+ = 0.091, SE E+ = 0.010, df E+ = 66. All linear regression slopes were statistically different from zero (data not shown).

Microscopy analyses demonstrated that E+ individuals were progressively colonized by DSE both extra and intracellularly. Considering that at the beginning of experiments there was no evidence of root colonization, the root infection process was successfully (Data not shown). By the end of the N-mineralization experiment (60 days), the percentage of infested roots in *C. quitensis* inoculated with *P. chrysogenum* reached 88.5 ± 1.6% and was 91.2 ± 0.9% in *D. antarctica* plants inoculated with *P. brevicompactum*. Relative to the temporal dynamic of the available NH_4_^+^ in the substrate of the experimental plants, GAMM models revealed for *C. quitensis* and *D. antarctica* a significant increase in time among the rhizospheric soil of both E− and E + plants (Supplementary Table 1). However, despite this general increase among all experimental groups, there was a significant influence of the infection status in both species, and particularly in *C. quitensis*, where E+ plants showed greater contents of NH4+ in their rhizospheres if compared with E− individuals (Figure 2). This can be easily observed in the absence of confidence interval overlapping in *C. quitensis*. By contrast, in *D. antarctica* the fitted splines for E+ and E− individuals appear close to each other, such as to do not appear statistically different in some time periods toward the end of the experiment (Figure 2).

**FIGURE 3:**
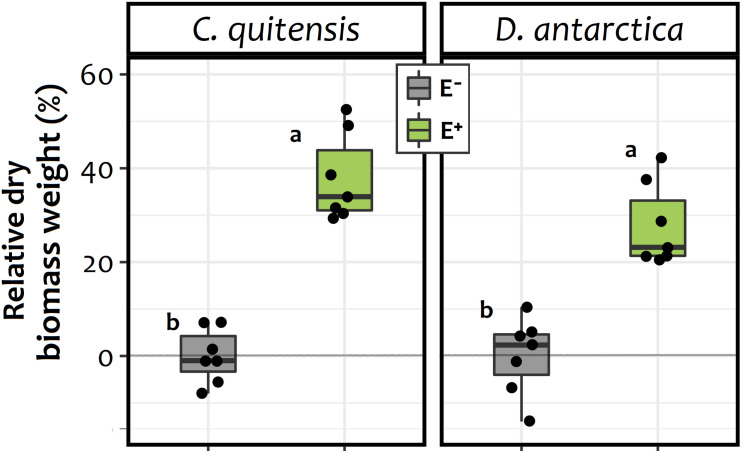
Biomass increase percentage of endophyte free (E−, in gray) and endophyte-infected (E+, in green) individuals of *C. quitensis* and *D. antarctica*, expressed as the percentage of the average final dry biomass of the E− group of each species. Dots represent individual values (*n* = 7), while boxplots the data inter-quartilic distribution per group. Different letters denote significant differences (*p* < 0.05) between treatments and/or species, as determined by the Tukey a-posteriori pairwise comparison test.

For both species, the enhanced availability of inorganic N in the form of NH_4_^+^ in soil of E+ individuals may explain their higher average dry biomass at the end of the experiment relative to E− plants (Figure 3). In this sense, a significant biomass increase of 34 and 23% was found for both *C. quitensis* and *D. antarctica* in E+ individuals, relative to their respective axenic E− counterparts [endophyte treatment: *F*_(1, 24)_ = 114.12; *p*≤ 0.0001]. However, there was no significant interaction between endophyte treatment and species, meaning that the effect of endophytes on plant biomass was similar in both *C. quitensis* and *D. antarctica* (Figure 3).

**FIGURE 4:**
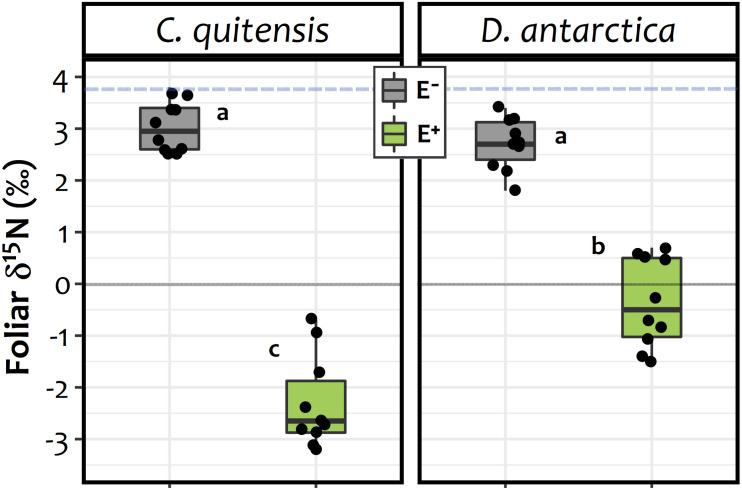
d15N signatures observed on the foliar tissues of *C. quitensis* and *D. antarctica* growing without fungal endophytes (E−, in gray), or inoculated with their most abundant root endophyte (E+, in green). Dots represent individual values (*n* = 10), while boxplots the data inter-quartilic distribution per group. Different letters imply significant differences (*p* < 0.05) between treatments and/or species, as determined by the Tukey a-posteriori pairwise comparison test. The blue dashed line denotes the isotopic signature of the initial substrate (d15N = 3.7‰). The zero represent outcome among the enrichment / depletion of the 15N isotopic, relative to the standard (i.e., the atmospheric 15N/14N ratio).

In relation to the ^15^N isotopic signature of the foliar tissues, significant differences were found between experimental groups (E+ and E− plants) in both species (Figure 4). The average δ^15^N values obtained showed that, relative to the isotopic fractionation in the initial substrate (δ^15^N = 3.67), the foliar tissue of *C. quitensis* and *D. antarctica* individuals from both endophyte treatments were depleted in ^15^N. However, the fractionation among E− plants (*C. quitensis*: 3.05 ± 0.51‰; *D. antarctica*: 2.71 ± 0.49‰) was far lower than in E + plants (*C. quitensis*: −2.31 ± 0.89‰; *D. antarctica* −0.35 ± 0.86‰). This suggests that for both species, the inoculated root endophytes were significantly involved in the process of N-uptake. Furthermore, the interaction term in the two-way ANOVA was statistically significant [endophyte treatment × species: *F*_(1, 36)_ = 25.27; *p* < 0.0001] with N fractionation being significantly greater in *C. quitensis* than in *D. antarctica*, but only in E+ plants (Tukey test, *p* < 0.05; Figure 4).

## Discussion

Our results indicate that the presence of the studied root endophytes significantly favored organic N mineralization in the rhizospheric soil associated with Antarctic vascular plants *Colobanthus quitensis* and *Deschampsia antarctica*. Additionally, endophytes favor N-uptake independently of the availability of NH_4_^+^ –an inorganic and easily assimilable N-source– in both species. Previous studies on Antarctic vascular plants have shown their capacity to modify the quality and composition of the soil organic N pool (Roberts et al., 2009), and their potential to obtain free amino-acids and small peptides from the soil (Hill et al., 2011, 2019). However, it has been recently demonstrated, at least for *D. antarctica*, that much of these rhizospheric dynamics of N-transformation and uptake in this plant rely on the activity of their fungal root endophytes (Hill et al., 2019). In this sense, the present results are complementary to those of Hill et al. (2019), who demonstrated the participation of endophytes in the uptake of small peptides by *D. antarctica* under controlled conditions. The greater accumulation of NH_4_^+^ in the soils of E+ plants of both species found in our study suggest that, together with their capacity to metabolize amino-acids in an early stage of organic matter decomposition, the rhizospheric mineralization of organic N forms like urea is also enhanced by root fungal endophytes. Nevertheless, increase of NH_4_^+^ over time in soil with plants (E−) also occurred, that could be explained by direct hydrolysis of urea in the soils or even as result of the mineralization performed by plants itself. Urease activity has been reported for other root endophytes (Jumpponen et al., 1998; Narisawa, 2017), and it is likely that the species used in this study also have the same metabolic capability. On the other hand, the improvement in the mineralization of the organic N source by endophytes could be related to the higher biomass found in E+ individuals relative to E− plants at the end of the experiment. Even though it is not possible through our experimental design to define which specific N uptake pathway was favored by the fungal symbiont, its presence definitively promotes the incorporation of N into the plant hosts. However, of all the possible N forms, NH_4_^+^ is the most plausible compound incorporated by these Antarctic plants after endophytic mineralization from urea.

Fungal endophtytes could also explain the higher N uptake efficiency that has been observed in Antarctic vascular plant species, particularly *D. antarctica*, when the inorganic N availability increases in the soil (Rabert et al., 2017). Indeed, the preference of *D. antarctica* for NH_4_^+^ as its main N source has been demonstrated, even when other inorganic N forms like NO_3_^–^ were available in a wide range from low- to high-levels (Rabert et al., 2017). In contrast, *C. quitensis* did not show any substrate preference when exposed to similar concentrations of these inorganic N compounds (Rabert et al., 2017). This may explain the pattern of NH_4_^+^ accumulation in soils found in this study, which was more evident in *C. quitensis* than in *D. antarctica.* Moreover, NH_4_^+^ accumulation in soils was higher in *C. quitensis* toward the end of the experiment (days 30 and 60) compared to *D. antarctica*. Thus, the higher efficiency of *D. antarctica* in acquiring NH_4_^+^ could explain the lower accumulation of this substrate in the soil, even under the improved mineralization promoted by the fungal inoculation.

It is important to highlight that the presence of root endophytes significantly changed the ^15^N isotopic signature in the foliar tissues of both species, demonstrating the active participation of this endophytic fungi in the process of N uptake by the host plant roots. Despite the ^15^N signature found in leaves of E+ and E− plant tissues from both species appear to be depleted relative to the substrate, this effect was significantly larger in leaves of inoculated (E+) individuals, particularly in *C. quitensis*. The slightly depleted, and still positive, ^15^N signal observed in the foliar tissues of E− plants is consistent with plants being grown on a ^15^N-enriched substrate, which is typical of ornithogenic soils (Zhu et al., 2009). This is because the process of ammonia volatilization that occurs spontaneously in the presence of water after an input of uric acid in the soil, strongly discriminates against the heavier N isotopes, increasing its proportion in the soil substrate as the lighter isotope leaves the soil pool as volatile NH_3_ (Erskine et al., 1998; Bokhorst et al., 2019b). For this reason, among E− plants, which acquire N without the aid of microbial symbionts, the isotopic signal in their tissues was similar to those of the substrate. Contrastingly, infected individuals (E+) of both species showed a negative isotopic ^15^N signature, indicating a larger depletion of the heavier isotope in the assimilated N, presumably by the N-fractionation generated by the fungal symbiont. This mineralization process, which should be analogous to those exerted by mycorrhizal fungi in Arctic plant species, produces ^15^N-enriched fungal tissues, while transferring ^15^N-depleted nitrogen forms to the plant host (Hobbie and Colpaert, 2003; Hobbie and Högberg, 2012). This would suggest that the δ^15^N signature in the endophyte biomass should also be enriched in ^15^N. However, due the anatomical distribution of the fungal endophytes inside the root tissues, it was not possible for us to measure this signature in the fungal biomass.

Several studies have estimated the proportion of N isotopes among the Antarctic biota, highlighting the role of marine-derived N on the fertilization of terrestrial ecosystems in relation to their proximity to active mammal and bird colonies (Erskine et al., 1998; Park et al., 2007; Bokhorst et al., 2019a,b). Nevertheless, these values could be highly variable depending on the local conditions. For example, Park et al. (2007) reported in the surroundings of Palmer station in Biscoe Point δ^15^N values of 11.2 and 11.0‰ for *C. quitensis* and *D. antarctica*, respectively, which showed also a small depletion in ^15^N respective to a 13.4‰ found in the soil (Park et al., 2007). However, in a similar study, Lee et al. (2009) found that the ^15^N isotopic signatures of *D. antarctica* from Barton peninsula (King George Island) varied between 0.4 and 4.5‰, depending on how influenced the plants were by the local bird nesting sites. In the light of this, the isotopic signatures found here appear particularly depleted in ^15^N (negative values for both species) probably because in our experimental setup we did not reproduce the continuous input of enriched ^15^N produced by animal colonies in the field and because the experimental soil was retrieved from a zone without marine animal influence.

It is important to acknowledge that experimental and laboratory conditions are drastically different from the field. For example, by accelerating the rate of N uptake process because growth chambers cannot mimic the exact interaction between temperatures, relative humidity, and radiation experienced by plant in natural conditions. Nonetheless, this do not override the positive effect of fungal endophytes in process uptake here. Similar to the plant-mycorrhiza model, a depleted isotopic signature in the leaves is a clear evidence of the fungal symbiont mediation in the N assimilation by the Antarctic host plants. However, our results suggest that the effect of fungal endophytes for N uptake is most pronounced for *C. quitensis* than for *D. antarctica*. This is because despite δ^15^N of foliar tissue in both species was significantly depleted relative to their E− counterparts, the fractionation between the substrate and the foliar tissues was lower in E+ plants of *D. antarctica* (δ^15^N_fract_ = 4.02), than E+ plants of *C. quitensis* (δ^15^N_fract_ = 5.98). It has been demonstrated that *D. antarctica* has the metabolic capability to incorporate small organic N-forms like amino acids and short peptides directly from the soil (Hill et al., 2019); a process that seems to fractionate less against the heavier isotope than the endophytic fungi does, leaving a less depleted signature in the plant tissue. However, this was not assessed in this study. Further research is needed to fully understand how fungal symbionts module different pathways of N acquisition and their relative relevance for each Antarctic vascular plant species.

Among cold environments the genus *Penicillium* has been observed in soil permafrost and ice-caps (Gunde-Cimerman et al., 2003; Zucconi et al., 2012). But it is also present in different Antarctic substrates such as oligotrophic (Godinho et al., 2015), ornithogenic (McRae et al., 1999), and the active layer of soil permafrost, in which spores of the two species studied here were present (Kochkina et al., 2014). In addition, some *Penicillium* species were found in different tissues of the Antarctic flora, including rhizoids of the liverwort *Cephaloziella varians* (Newsham, 2010) and shoot of the moss *Bryum argenteum* (Bradner et al., 2000). Nevertheless, has been poorly demonstrated the role of fungal endophytes (e.g., *Penicillium* spp.) in the nitrogen uptake assessed by the isotopic modulation and/or fractionation rates.

**FIGURE 5:**
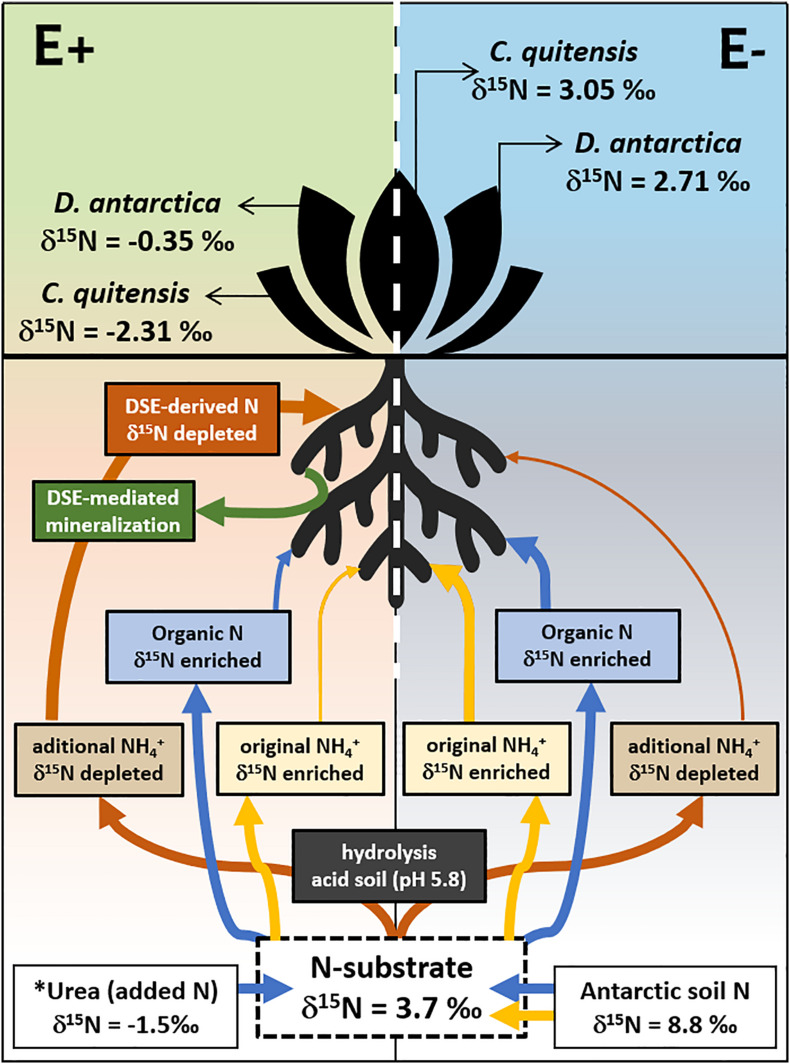
Proposed model of the DSE-Plant interaction for N uptake in the vascular Antarctic plants *Deschampsia antarctica* and *Colobanthus quitensis*, determined by the d15N signature in soil and leaves. E denote non-infected plants while E+ their DSE-infected counterpart. Arrow’s width imply preferred N-substrates uptaked/assimilated while their color represents its form in the soil (blue: organic; red/yellow: inorganic). The proportion of the urea added (8 g/kg) was equivalent to the previously estimated total N content of the experimental substrate (see methods for details). ^∗^ Note that the urea added to the incubations are much more 15N depleted than the Antarctic soil used. Eliminado: Mineralization of organic N as described by the accumulation of NH4+ in the substrate of endophyte free (E−, in gray) and endophyte-infected (E+, in green) individuals of the two Antarctic vascular plant species. Boxplots represent the inter-quartilic distribution of the data (*n* = 7), different letters denote significant differences after a factorial pairwise comparison using Estimated-Marginal Means (EMMs) analysis with a 0.95 confidence level.

Based on our experimental results, we build a conceptual model (see Figure 5) that illustrates the effects of DSE in the nutrient acquisition in the two native vascular Antarctic plants. In the absence of DSE endophytes, E− plants seem to mainly uptake enriched N-sources, either from the enriched NH_4_^+^ previously present in the field soil samples, or from the small organic compounds (e.g., amino acids and short-chain peptides) that Antarctic plant species may be capable to uptake (Hill et al., 2019). A proportion of the urea-derived NH_4_^+^ (δ^15^N -1.5‰), which hydrolyzed spontaneously at the acidic conditions (pH 5.8), found in the soil, could also be uptaken due to the high affinity of plants for this N form, particularly by *D. antarctica* (Rabert et al., 2017). However, despite the presence of this ^15^N-depleted NH_4_^+^ source in the substrate of all experimental plants, the ^15^N signature in the final tissues of E− plants from both species was less fractionated (δ^15^N 2.7–3.1‰), yet, partly depleted relative to the soil substrate (δ^15^N 3.7‰). By contrast, the symbiotic interaction left a signature in the foliar tissues of E+ plants that was far more ^15^N-depleted (δ^15^N −0.4 to −2.3‰) than E− plants relative to the ^15^N in the initial substrate, such as has been previously proposed (Hobbie and Högberg, 2012; and references therein). In this sense, this isotopic signature strongly suggests that a large proportion of the N taken up, is preferentially managed through endophytes-mineralized N compounds, probably in the form of NH_4_^+^. This may be supported by the higher mineralization registered in the soils from E + individuals from both species compared with their axenic counterparts.

## Conclusion

In conclusion, here we corroborate that despite being grown under rich N soils, DSE exert a positive effect in the N-uptake of the two Antarctic vascular plants. This effect was mediated both, by the enhanced availability of inorganic N sources in the substrate such as NH_4_^+^, but also by the active participation of fungal endophyte in the process of N-uptake, as suggested by the isotopic signature encountered in the foliar tissues of these plant species. Although, further research is needed to determine the specific routes by which fungal endophytes fulfill this role, here we identify some promising avenues of research to accomplish such a goal.

## Data Availability Statement

The raw data supporting the conclusions of this article will be made available by the authors, without undue reservation.

## Author Contributions

IA-R, CT-D, and MM-M designed and performed the experiments. IA-R, AG, and CA analyzed the data. All authors wrote and reviewed the manuscript.

## Conflict of Interest

The authors declare that the research was conducted in the absence of any commercial or financial relationships that could be construed as a potential conflict of interest.

## Footnotes

1 http://biologia.utalca.cl
