# Supplementary material for: Fungal Symbionts Enhance N-Uptake for Antarctic Plants Even in Non-N Limited Soils
Source: Front Microbiol. 2020 Oct 23;11:575563. doi: 10.3389/fmicb.2020.575563 (PMC7645117; doi:10.3389/fmicb.2020.575563)

*Deschampsia antarctica* and *Colobanthus quitensis* with (E+) and without (E-) presence of root fungal endophytes. The occurrence of effective symbiosis was corroborated by routine staining and microscopic observation in three randomly selected individuals from each species.


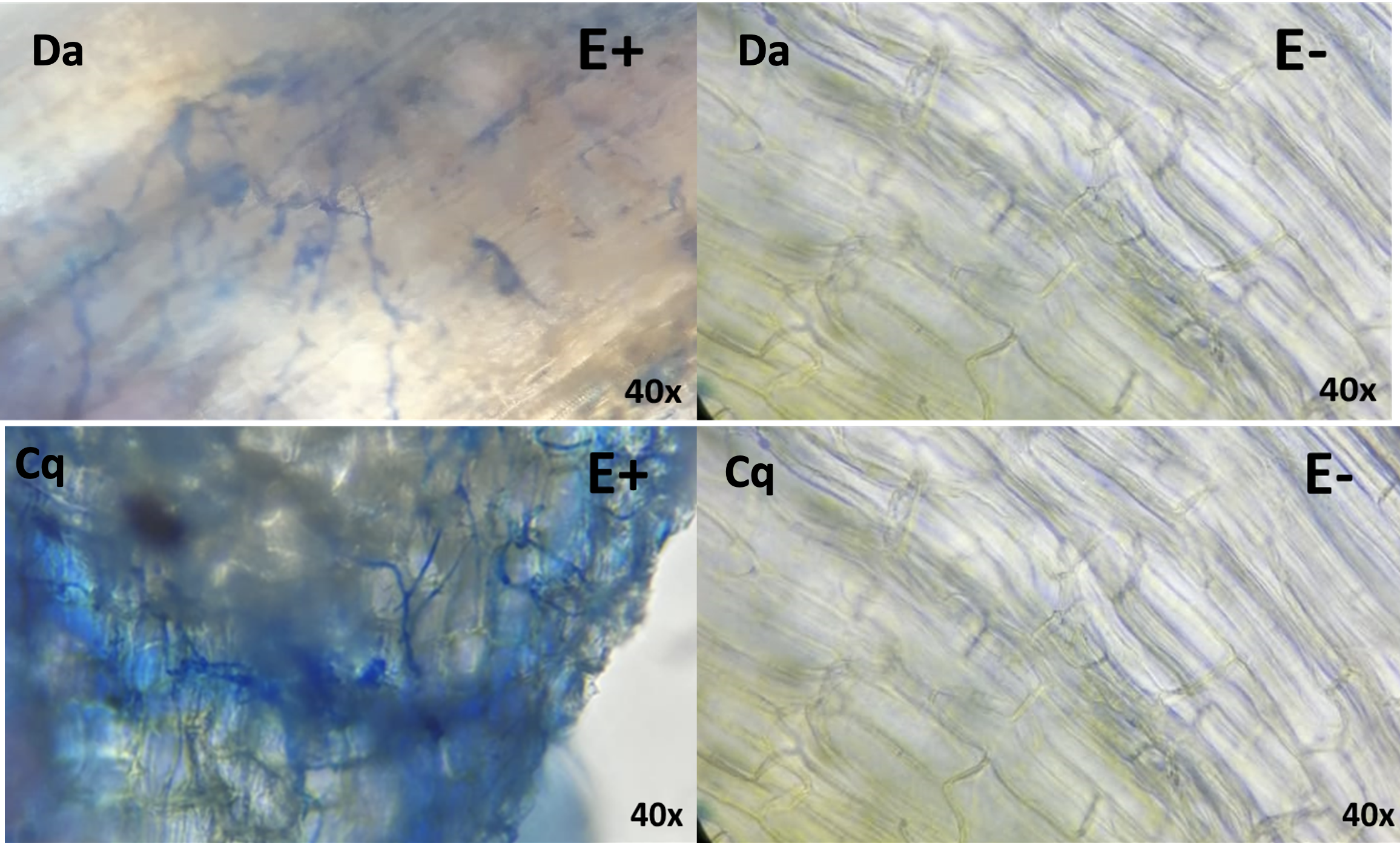

Supplement: Supplementary file 1 [file Data_Sheet_1.DOC]
