# Supplementary material for: Fungal Symbionts Enhance N-Uptake for Antarctic Plants Even in Non-N Limited Soils
Source: Front Microbiol. 2020 Oct 23;11:575563. doi: 10.3389/fmicb.2020.575563 (PMC7645117; doi:10.3389/fmicb.2020.575563)
Supplement: Supplementary file 2 [file Table_1.DOC]

**Supplementary Table 1:** Approximate significance of the temporal trend denoted by the edaphic NH_4_^+^ in the rhizosphere of both, endophyte-free (E-) and infected (E+) experimental plant individuals. The smoothed function was fitted by species using a Generalized Additive Mixed Model (GAMM) approach. Red *p*-values represent significant reductions in the model deviance due to the inclusion of the respective splined term. The overall goodness-of-fit for each species’ model is described by the adjusted *R^2^* term below their corresponding names.

| **Plant**  **species** | **Infection**  **status** | **estimated**  **d.f.** | ***F***  **statistic** | ***p***  **value** |
| --- | --- | --- | --- | --- |
| ***C. quitensis*** | E- | 3.32 | 64.12 | < 0.0001 |
| (*R^2^_adj_* = 0.94) | E+ | 4.04 | 174.33 | < 0.0001 |
| ***D. antarctica*** | E- | 2.77 | 48.83 | < 0.0001 |
| (*R^2^_adj_* = 0.86) | E+ | 3.57 | 67.88 | < 0.0001 |
